# Feeling known and informed: Serial qualitative interviews evaluating a consultation support intervention for patients with high‐grade glioma

**DOI:** 10.1002/cam4.5572

**Published:** 2023-01-17

**Authors:** Sarah C. Shepherd, Belinda Hacking, Louise M. Wallace, Sarah E. Murdoch, Jeff Belkora

**Affiliations:** ^1^ Division of Medical Education University of Manchester Manchester UK; ^2^ NHS Lothian Edinburgh UK; ^3^ Faculty of Wellbeing, Education & Language Studies The Open University Milton Keynes UK; ^4^ Public Health Scotland Edinburgh UK; ^5^ Surgery and Health Policy University of California San Francisco (UCSF) San Francisco California USA

**Keywords:** cancer, decision support, high grade glioma, oncology, patient involvement, patient participation, patient–physician relations, qualitative research, self‐efficacy, shared decision making

## Abstract

**Objective:**

Interventions to support patients' engagement in shared decision making (SDM) are lacking within high‐grade glioma (HGG) healthcare. Consultation Planning, Recording and Summarising (CPRS) has shown evidence of increasing patient decision self‐efficacy, reducing uncertainty, and regret of decisions. This is the first study of CPRS within a HGG population and delivered over serial medical consultations.

**Method:**

A one‐arm prospective qualitative longitudinal design was used to evaluate the CPRS intervention and evaluated with participants at sequential clinic appointments depending on their care, in Edinburgh, Scotland. We report on serial semi structured interviews of 16 patients and their partners.

**Results:**

Consultation planning before the consultation supported patients to feel known by strengthening the patient voice within the consultation. It prepared patients to actively participate in the consultation, despite the distressing nature of the content. Recording and summarising supported patients to understand their situation. The provision of a consultation record enabled accurate recall, a paced uptake of information and supported the family to feel fully informed. Ultimately, patients understood why decisions were being made rather than being part of making decisions.

**Conclusions:**

The CPRS intervention helped patients to understand and to feel known by increasing patient capacity for communication in the consultation, with support before, during, and after the consultation. The intervention focused on preparing patients for SDM but patients did not perceive that they had meaningful choices to make. Further research could look at the inclusion of patient decision aids to support this process.

## Graphical abstract

This study illustrates how Consultation Planning, Recording and Summarising (CPRS) may help people with HGG to understand and feel understood as they navigate their healthcare from diagnosis. Consultation planning amplified the patient voice, helping patients feel understood. The recording and summarising, amplified the physician voice, helping patients understand.

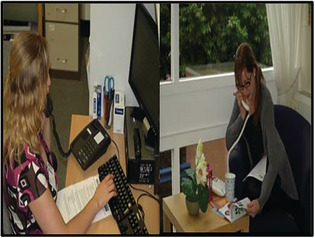

## 1 BACKGROUND

High‐grade glioma (HGG) is a rare cancer with no current curative treatments.^1^ As treatment options are limited and with palliative intent, aspects of care such as communication and support practices are crucial.^2^ The uncertainty of treatment outcomes underpins the importance of involving patients in care decisions. However, there is sparse research on shared decision making (SDM) in this patient population. Shared decision making occurs when clinicians and patients work together to decide a way forward based on clinical evidence and patient values.^3^

A recent systematic review into treatment decision making found that SDM was important to this HGG population.^4^ However, this review is based on only four eligible papers, and only one that studied the HGG population exclusively.^5^ This small sample (*n* = 26) survey study found lower levels of anxiety in patients who wanted, comprehended, and were satisfied with the information provided at the point of decision making about surgery.^5^

Given a core component of UK policy for improving healthcare outcomes is the empowerment of patients through information provision to enable engagement in shared decisions with clinicians, this paucity of research represents a major gap in knowledge for this population.^6^

This small observational study also found HGG participants with greater information satisfaction experienced less anxiety, measured by the Hospital Anxiety and Depression Scale.^5^ This finding is supported by two qualitative studies exploring the lived experience of HGG^7^, ^8^ which observed appropriate information reduced anxiety. In the first study, information needs varied between and within patients and as the disease progressed.^7^ Providing both verbal and written information supported patients to understand and pace information.^7^ In the second study, when HGG patients felt “left in the dark” a sense of uncertainty arose, creating space for negative predictions of the future.^8^ Patients were often so traumatised by the diagnosis and they were unable to retain information.^8^ Both studies found engaging in shared decision making (SDM) was difficult for participants to achieve due to the uncertainty of the disease progression and lack of viable treatment alternatives.^7^, ^8^

A promising intervention to support SDM is consultation planning, recording, and summarising (CPRS). It comprises three evidence‐based interventions; coached question listing^9^ followed by audio recording and summarising^10^ of the consultation by a non‐clinical navigator to provide a communication support tool for patients. In trials, CPRS has been found to be feasible and effective in two UK oncology populations supporting key treatment decision making points, increasing decision self‐efficacy and reducing decision conflict^11^, ^12^, ^13^ and has a strong evidence base of facilitating communication and decision making in cancer consultations in the United States.^14^, ^15^, ^16^ It has not been evaluated with a HGG population. The tailored and interactive nature of CPRS enables it to be a useful tool when a patient may require support in engaging with a consultation. This study uniquely adds to the body of CPRS evidence in two ways; (1) supporting HGG patients, and (2) providing the intervention over consecutive clinic appointments. As such, this study aims to evaluate for the first time the experience of serial use of CPRS for newly diagnosed HGG patients through their first line treatment.

The theoretical framework that informed this study is the model of the medical encounter proposed by Bensing and Verhaak.^17^ This model suggests that when faced with illness there are two needs: the need to understand the disease and to feel understood as a unique person. It is hypothesised that the CPRS intervention may address both needs; the need to understand, and the need to feel understood.

## 2 METHOD

### 2.1 Study design

A prospective qualitative longitudinal design was used to evaluate the CPRS intervention, delivered by one of two trained non‐clinical navigators to study participants at their sequential clinic appointments. Interviews were conducted after consultations.

### 2.2 Setting

A neuro‐oncology clinic in Scotland (UK) between January 2011 and March 2014. Ethical approval was obtained from South East Scotland Research Ethics Committee (10/S1103/47), Research and Development, Queens Medical Research Institute, (2010/W/ON/19) and Coventry University (17/09/2010).

### 2.3 Participants

Eligible participants were aged over 18 undergoing investigations for suspected primary malignant HGG. Participants were excluded if they were not able to converse in English and/or with their healthcare team deemed them too unwell or with diminished cognitive capacity.

### 2.4 Recruitment

Patients were invited to the study at the pre‐assessment clinic before their surgery or in the neuro‐surgical ward before or after their surgery. Eligible participants were identified through the weekly multi‐disciplinary team meeting and confirmed by the neuro‐surgical team. Eligible participants were offered the study by the healthcare team and consent was taken by the research team. The consent process focused on the patient as the active recipient of the intervention. When patients wanted caregivers involved, they were included them in the interviews/planning however, the patient experience/questions remained the focus of the study.

### 2.5 Intervention

The CPRS was implemented following a protocol described in detail in a case report.^15^

## 3 THE INTERVENTION

### 3.1 Navigators

Each participant was assigned a Navigator who delivered the intervention [steps A and B below] at up to four clinic appointments. In this study both Navigators were research psychologists. To standardise the delivery of the intervention, the navigators followed the protocol and training programme developed by author JB.^14^ The training included 4 h didactic material, 4 h intensive role plays, and a supervised practice and feedback with patients not included in this study. In addition, Navigators spent time observing neuro‐oncology clinic appointments to become familiar with the nature of the consultations and the clinic teams.

The intervention steps included:

- *Consultation planning (CP)*: Prior to the below clinic appointments, participants and their navigator generated a Consultation Plan (CP) containing personal questions, concerns, and key information for their next medical consultation. This was facilitated by a CP prompt sheet.^14^ The CP was shared with the patient and clinician before the appointment and a printed version was provided at the appointment. The planning appointments were not timed; however, the navigators reported an approximate range of 20–40 min per consultation planning. Table 1 provides themes of frequently asked question during consultation planning per clinic appointment.
- *Summary and audio recording*: The navigator attended the below clinic appointments with the participant to type notes and audio record. Participants received the audio recording of their consultation via CD immediately afterward. The navigator provided a plain language typed summary of their notes, approved by the attending clinician, within 1 week.

**TABLE 1:** Examples of frequently asked questions at each appointment

Apt.	Questions requesting clarity on:
1	Current symptoms and current medications Pathology/test results Treatment plan What I can do e.g., holiday, exercise, work, driving, intimacy, hair dye Prognosis Need for further support e.g., psychological, financial, information
2	Symptoms Medication Results/Treatment efficacy Next steps Frequency of follow ups Quality of life e.g., exercise, holiday, work, what should I/shouldn't I be doing
3	Changes in scan results/treatment efficacy Future treatment options Medication Follow‐up How to recognise recurrent Quality of life e.g., what does future hold, expected quality of life, psychological/financial support, support outside the hospital

## 4 CLINICAL APPOINTMENTS WITH CPRS

The following appointments were included in the intervention, but not all patients needed all four appointments.

1. *Initial diagnosis consultation*: At this appointment patients received their formal diagnosis following surgery. This appointment confirmed a diagnosis of high‐grade glioma [HGG]. Within this appointment patients were offered the treatment options of radiotherapy, with or without chemotherapy and the inclusion into two clinical trials (this varied throughout the study timeline).
2. *Three‐month follow‐up appointment*: At this point a patient's scan is reviewed to see if it indicates progression, stability, or reduction in the tumour. The result of this scan guided the next steps of treatment if any are deemed suitable.
3. *Consultation following chemotherapy treatment [only for participants who had chemotherapy as part of their treatment regime]*: At the end of chemotherapy patients attend this consultation to discuss management of chemotherapy side effects, possible future treatment options, and signs of progression to look out for.

### 4.1 Interviews

To evaluate the intervention, participants were invited to take part in three semi‐structured interviews by telephone, in person at the hospital, or at participants' home with author SS. Interviews were conducted alongside the intervention and arranged at the earliest convenience for the participant. A Telephone interview as an alternative to in person was offered to support participation in the study and reduce study burden. The Ottawa decision support framework^18^ guided the interview schedule. The questions examined the participant's knowledge, information needs, support received, expectations, and role in treatment decision making. Participants were able to include family perspectives as this approach appears not to inhibit data collection about sensitive issues.^19^ The researcher noted the partners' views as they reflected on the intervention and so their perspective was included in the analysis.

Interviews were conducted in parallel with the clinic appointments:

1. Before clinic appointment one [prior to any intervention]
2. Following the second clinic appointment
3. After the third clinic appointment if no chemotherapy, or after fourth clinic appointment if chemotherapy included in treatment regime [i.e., once no active treatment being received].

### 4.2 Data analysis

Interviews were conducted by one researcher (SS), audio recorded and transcribed verbatim. Framework analysis^20^ was used to analyse the data. This type of analysis was chosen as it provides coherence and structure for a focused exploration of issues determined to be of interest prior to the study. There were five key stages (1) Familiarisation with the contents of the transcripts; (2) a thematic framework was developed—informed by the theoretical framework—identifying key themes emerging and testing until a good fit was generated; (3) an index of the themes was transposed onto each transcript; (4) charts per theme were then created using quotes from transcripts. For quality assurance, a second researcher viewed the data and rated the match between quote and description of the theme, and (5) a narrative from the data was generated.

Inter‐rater reliability was checked during stage three of the analysis process [indexing] a 5‐page transcript from two different interviews was provided to an independent researcher. Any discrepancies were discussed, and the framework evolved to robustly guide stage four of the process [charting].

The interviews in this study aimed to evaluate the intervention in the context of the HGG participants' life. As such, the interviews gathered details about a specific and consistently delivered intervention. Saturation was considered reached when participants both within the two interviews and between each other were consistently repeating the same themes in reflection of their experience of the intervention, and no new fully formed themes were emerging. When planning for saturation a pragmatic approach was needed considering the research burden on this cohort.

## 5 RESULTS

### 5.1 Patient sample

**FIGURE 1:**
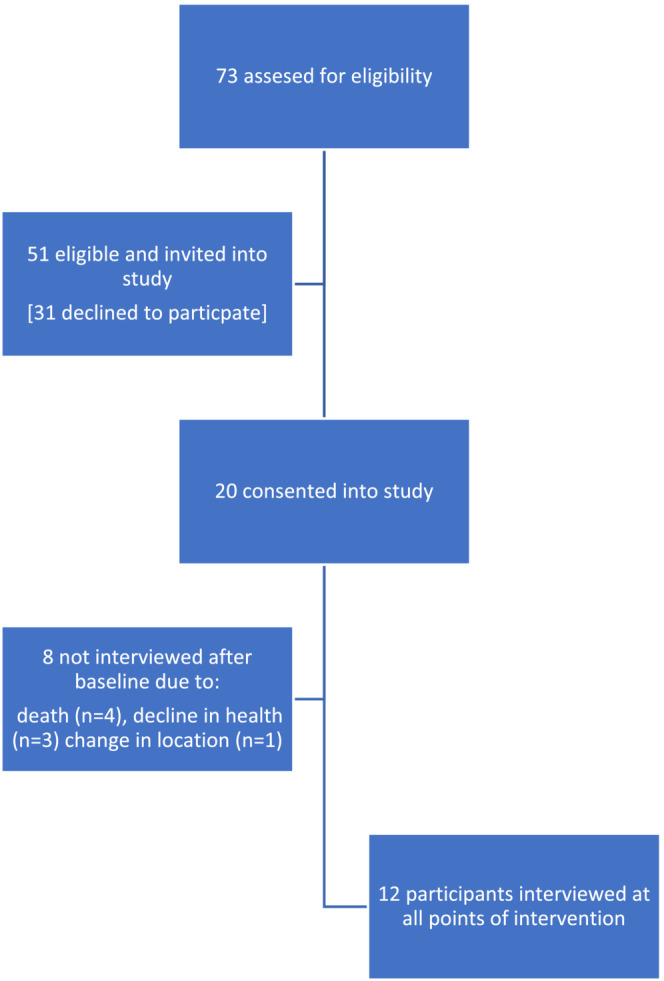
Flow chart of consent in study.

Seventy‐three participants were assessed for eligibility, 51 were invited to participate, 20 consented, see Figure 1.

Eight of the original cohort of 20 were not interviewed after baseline due to; death (*n* = 4), experiencing a physical or mental decline (*n* = 3), or due to re‐location for further care (*n* = 1).

Twelve participants were interviewed three times – however, this paper focuses only on the interviews which evaluated the intervention. As such, this study reports on the final two interviews for twelve participants. Four of the twelve participants preferred paired interviews with their husband (*n* = 2) or wife (*n* = 2) so, 16 people (12 participants and four partners) were interviewed twice, generating 24 interviews, see Table 2. The mean age of the participants interviewed at T2 and T3 was 49.75 years, seven were female. Most (*n* = 10) were diagnosed with a glioblastoma (GBM), see Table 2.

**TABLE. 2:** Characteristics of participants interviewed

Participant name	Paired or single interview	Gender	Age	Diagnosis	Navigated appointments	Location of interviews
A	Single	Male	53	GBM	3	Phone
B	Single	Female	30	GBM	3	Face to face
C	Single	Female	29	GBM	4 [extra initial appointment]	Phone
D	Paired	Female	57	GBM	3	Face to face
E	Single	Female	39	Anaplastic Oligodendroglioma	3	Phone
F	Paired	Female	59	Anaplastic Oligodendroglioma	2	Face to face
G	Single	Male	69	GBM	3	Phone
H	Single	Male	68	GBM	3	Phone
I	Single	Male	47	GBM	4 [extra initial appointment]	Face to face
J	Single	Female	34	GBM	3	Face to face
K	Paired	Male	56	GBM	3	Face to face
L	Paired	Female	56	GBM	3	Face to face

Most participants (*n* = 9) were navigated three times, see Table 2. For patient privacy, and to avoid using pseudonyms that are culturally biased, we refer to the patients alphabetically as Patient A, etc. Patient C and I requested an extra appointment following their diagnosis to further discuss treatment and so received an extra dose of the intervention. A break in protocol occurred for Patient F as no Navigators were available to attend her mid‐treatment appointment due to illness, they did not receive an audio recording and summary for this appointment.

## 6 FINDINGS

Using the theoretical framework,^17^ the themes were organised into how the intervention enabled patients' affective need to feel known and cognitive need to know and understand.

### 6.1 Enabling the patient to feel known and understood

#### 6.1.1 Preparing the patient to express their questions and concerns

There is a profound difference between saying what you are thinking and actively creating a message for the receiver to understand. Creating a clear message becomes harder when the topic is so distressing. Consultation planning (CP) provided a structured space for participants to express and formulate their thoughts, feelings, and questions without the distraction of premature answers or advice. During CP, navigators engaged in deep active listening and created neutral and patient centred summaries.

‘It is helpful to formulate what you want to ask beforehand or, you know, have some help to do that.’ Patient E T2

On that day (appointment 1) I don't think I would have been able to structure my thoughts and my questions and ask them without having them written down in front of me, and without Dr (name) having them …[ ]… I just wouldn't have been able to do it without the support of the navigator. Patient I, T2

The process of talking everything through not only helped in formulating information and questions, it also encouraged participants, and often their partners, to focus on and think about what it was they wanted to know. This was not an easy task when the content meant confronting a future that presented significant existential concerns.

It's like, you're thinking about it a little bit more, rather than just going in there and feeling that the questions are then building up in my head again. Patient C, 29, T2

The project forced us in advance of our consultation appointments each time to think about the things that we only survive by not thinking about everyday… Partner of F T3

The most important thing is the fact that, you know, any questions you've got …. you come out with them. So that's vital. Because if I'm honest, when you go in that room the only thing you're thinking about is, “How did the scan go?” Partner of D T3.

#### 6.1.2 Creating space for the patient voice in the consultation

Supported by their CP appointment participants felt confident to share and gather information in the consultation without being dependent on memory or their ability to verbalise questions. Two participants described how the CP helped them remain present in the discussion about distressing issues in their diagnostic appointment. Participants attributed knowing they would have an audio and written summary of the consultation to them feeling more relaxed and focused within the consultation. The crucial timepoints for this intervention emerged as the beginning and end of treatment.

Going into the doctor's, there's a million other things you think about asking and you never actually do, so having them down in front of the doctor to start with is, like, it's just brilliant. Patient C, T2.

…because of the pre‐questions, I've asked what I wanted to ask. Patient G, 69, GBM.

I think the first was the most useful. The (Mid treatment), I would say it was still useful, but it was less so because I was much more aware of what was happening to me, and what my treatment was and when it was going to finish. But then the last one, I thought was useful again, because it helped to focus my attention on, well, what happens next? Patient I, T3.

### 6.2 Enabling the patient to know and understand

#### 6.2.1 Accurate information provision

The consultation summary enabled participants to check the accuracy of their recall, removing the need for follow‐up contact with healthcare staff for information. Participants used the audio file (CD) and summary as a memory aid, and this helped them to feel reassured they had remembered information accurately:

‘It helps you to recall just exactly what was said about any particular aspect and that's quite reassuring.’ Patient G, T3; (gather answers and clarify symptoms they were experiencing).

‘I felt really, really tired, probably about four to six weeks afterwards. And that's what they said would happen.’ Patient E, T3 (referring to checking the consultation summary).

If we think what was that again? We can always run through it. Patient K, 56, T3 (referring to the audio file).

When partners recall differed, they could use the summary to check and resolve any conflicts in understanding quickly, as well as pacing the processing of information.

‘Helps iron things out when two people have taken away differing impressions of what happened within the consultation.’ Partner of F, T3

‘When we read them over again, we realised there was bits that we hadn't quite taken in at that point or … that [we] had just kind of glossed over. So, … having that fuller set of information that was quite useful. Patient F, T3

Providing the doctor with the CP before the meeting seemed to override the unease that can manifest with seeing different doctors, as patients felt their doctors were more prepared for them.

Whichever consultant you see has that info, you know, as well, so that they are already preparing a full answer [to your questions]. Patient E, 39, T3.

Participants continued to use their summaries following the end of their treatment. This provided reassurance and courage in how they had managed to cope.

‘In some ways it was quite nice to read it all again and remind myself of, how difficult and painful it was but, I think that helped remind me that it was quite a long time ago and that I'm still here a year later and I'm feeling better, and I am better.’ Patient I, T3.

#### 6.2.2 Informed family members—‘they know as much as I know’

An accurate consultation record was reassuring for those close to the patient who were unable to attend the appointment and meant participants did not have to explain their situation repeatedly. Participants valued this and reported it had a calming effect, everyone knew what had been spoken about in the consultation:

‘The other benefit I have is my two children can listen to the tape or read the transcript and they've got first‐hand information. It's not what I remember to tell them, and I think that's a great thing for them as well. I feel quite relaxed that they know as much as I do.’ Patient G, T3

‘we've told her [daughter] how it went but I think for her to sit and listen to it is probably more beneficial. Patient L, T2

‘I think that [provision of summary] … calmed the whole family down a bit, because it's in black and white, written down for you’ Patient C, T3

#### 6.2.3 Written versus audio information

Most participants preferred the written summary over the audio recording [CD]. Locating pieces of information in the summary was much easier and faster this way. Listening to the recording, especially of their first appointment, was often too painful, however, two participants preferred to use the recording, particularly when the appointments were less emotive.

“it's quite unnerving to hear all that. It's raw to listen to the actual tape [CD], too raw.” Partner of F, T3.

“The last occasion – I just had [used] the CD rather than the actual, all the writing. Because with a CD you had all the information there that the doctor was saying to you.” Patient H, T3

### 6.3 Informed not shared decisions

Participants perceived decisions were made by their clinicians, appreciating that treatment plans were based on best practice clinical guidelines. Despite being actively involved in ensuring a high quality of information exchange and understanding of the information provided, participants did not feel they had a meaningful choice in their treatment. Evident in all narratives was the improbability of going against the doctors' recommendations. There was, however, reassurance and emotional benefit from understanding the rationale for the proposed treatment plan.

‘you're going to go with what they recommend I think, it's difficult not to.’ Patient G, T3

‘it's the clinical judgement they make for everyone. You know, “We're going to give you radiotherapy because it's the best treatment available.’ Patient I, T3 GBM

## 7 DISCUSSION

This study illuminates how communication aids may help people with HGG to understand and feel understood as they navigate their care. The first communication aid, consultation planning, amplified the patient voice, helping patients feel understood. The recording and summarising, amplified the physician voice, helping patients understand.

Consultation planning supported participants to generate important questions and information to share with their clinicians. Participants reported this helped them receive meaningful information from their clinician. Personalised information is an important aspect of care,^5^ supporting the feeling of “being known” ‐ an acknowledgement of the individual as a unique person, distinct from the disease.^21^

A unique finding was how consultation planning united patients and their partners to ensure a shared agenda before the consultation. Cavers et al.^8^ noted differences in information preferences between relatives and patients was often a source of tension and distress. This is complex as careers often have a greater wish for information than patients.^22^ In this study, patients and their partners felt supported to plan and negotiate their information needs in advance.

The provision of a consultation summary and recording reassured participants. Timepoints noted as key for the intervention were the initial and end of treatment appointments. Both verbal and written information was helpful and enabled paced information uptake,^4^, ^5^ preferable for HGG patients as they gradually adjust psychologically to their disease and prognosis.^23^ These experiences contrast with the HGG usual care experience defined by a lack of meaningful information.^4^, ^5^, ^22^

For SDM to be a reality, the focus is often placed on honing the clinician's skills. This study highlights patients also need to accomplish a wide variety of behaviours. A recent qualitative study illustrated this and proposed key elements are needed to enable patients to feel ready for SDM.^24^ The results from our study suggest the CPRS intervention attends to 3 of these key elements; (1) increasing self‐awareness of values, and (2) enabling patient's space within the consultation to articulate their feelings, identify their questions and express their values via consultation planning, alongside (3) increasing the health literacy of the patient by providing consultation summaries and audio recordings for patients to absorb the information despite the difficulty of the news.^24^

Although supported in their readiness for SDM, participants did not perceive having a meaningful choice to make. Communication aids may increase patient understanding and feeling of being understood and still not be sufficient to achieve all the conditions for SDM. Halkett et al.^7^ also identified that it was difficult for patients to be actively involved in decisions when they were reduced to treat or not treat, which is often the scenario for HGG patients. Physicians may require training on how to discuss the limited efficacy of aggressive treatments and the relative effectiveness of palliative care. In other areas of care, researchers have combined communication aids with decision aids. This has been effective in breast cancer care,^25^ orthopedics^26^ and further positive effects have come from combining coaching with decision aids.^27^ Decision aids often elevate the viability of active surveillance, or comfort care, or other modalities that may not be offered by specialists who naturally emphasise the treatments they are trained to provide.

It is important to acknowledge the challenges inherent with compressing complex decision making into a single brief consultation. In line with theories of quality improvement, the CPRS intervention transforms the event into a process^28^ by intervening before, during, and after the consultation. This extends the period of critical reflection, which otherwise tends to be compressed into an encounter where all parties may experience time pressure and other stressors. With CPRS, patients and family members take time in advance of the consultation to list questions with a navigator who proceeds at the pace of the patient. During the consultation, patients can consult their consultation plan to ask questions, and relax about the need to retain all the information, since they can rely on the recording and note‐taking services to capture it for later review. Afterwards, patients can review recordings and summaries and take time to absorb and to understand. Extending this period of critical reflection provides patients more time to formulate their reactions to their diagnosis; analyse the information; synthesise insights; and translate their insights into action. This conceptual model, known by its acronym as the FAST (Formulating and Analyzing issues, Synthesizing insights, and Translating the insights into action) process of critical reflection, is described in the literature^29^ and in a handbook for clinicians.^30^

### 7.1 Study limitations

Natural attrition resulted in a small sample size. However, data saturation was evident in analysis. With data collection limited to one tertiary site, findings are specific to these populations and transferability of findings may be limited to this context.

Further research may consider interviews with other data sources for example, evaluation from healthcare providers and using other data collection methods, example, field observation in ethnography, video recordings of the medical encounter, to provide a more comprehensive description of the social phenomena of decision making. When reflecting on the exclusions for this study further research should explore planned avenues of supporting those with diminished cognition or supporting their caregiver on their behalf.

### 7.2 Implications and final remarks

Overall, the CPRS intervention has the potential to be a powerful adjunct in the personalised care of HGG. Findings from this study have identified that all components of the navigation intervention were important and useful for participants and their relatives. This study is the first to provide understanding of how the unique combination of CPRS elements can enable and support HGG patients and their clinicians.

## CONFLICT OF INTEREST

The authors declared there are no conflicts of interest.

## Data Availability

The data that support the findings of this study are available on request from the corresponding author. The data are not publicly available due to privacy or ethical restrictions.
